# Carbapenem-resistant Enterobacterales among hospitalized patients in Cape Town, South Africa: clinical and microbiological epidemiology

**DOI:** 10.1093/jacamr/dlae051

**Published:** 2024-03-22

**Authors:** Hafsah Deepa Tootla, Elizabeth Prentice, Clinton Moodley, Gert Marais, Nyasha Nyakutira, Kessendri Reddy, Colleen Bamford, Abraham Niehaus, Andrew Whitelaw, Adrian Brink, Members (collaborators) of the Cape Town Antimicrobial Stewardship Study Alliance (CT-ASSA), Claudine Page, Elizabeth Schoeman, Elizma de Klerk, Karin Lategan, Karlien Pienaar, Liezl Henning, Mandy Du Plessis, Nomfundo Maseko, Salome Nel, Melenie Narainsamy, Michelle Vermeulen, Narissa du Toit, Teresa van Heerden, Liza Sitharam, Asa Barendse, Dane Nagel, Jacqueline Prince, Letitia Vass, Rileen Strauss, Rushana Fakier, Catherine Samuel, Marelieze van Zyl, Leigh-Ann Isaacs, Shareefa Hendricks, Amy Dodd, Reecka Daniels, Widaad Zemanay, Judi Van Heerden, Nchimunya Hapeela, Parveen Brown, Zubayr Daniels, Shantelle Claassen, Fadheela Patel, Sharon Vasuthevan, Enid Scott, Esmeralda Ricks, Patricia Curle, Justyna Wojno

**Affiliations:** Division of Medical Microbiology, National Health Laboratory Service, Red Cross War Memorial Children’s Hospital, Cape Town, South Africa; Division of Medical Microbiology, National Health Laboratory Service, Groote Schuur Hospital, Cape Town, South Africa; Division of Medical Microbiology, Faculty of Health Sciences, University of Cape Town, Cape Town, South Africa; Division of Medical Microbiology, National Health Laboratory Service, Groote Schuur Hospital, Cape Town, South Africa; Division of Medical Microbiology, Faculty of Health Sciences, University of Cape Town, Cape Town, South Africa; Division of Medical Microbiology, National Health Laboratory Service, Groote Schuur Hospital, Cape Town, South Africa; Division of Medical Microbiology, Faculty of Health Sciences, University of Cape Town, Cape Town, South Africa; Division of Medical Microbiology, Faculty of Health Sciences, University of Cape Town, Cape Town, South Africa; Division of Medical Microbiology, National Health Laboratory Service, Tygerberg Hospital, Cape Town, South Africa; Division of Medical Microbiology, Faculty of Medicine and Health Sciences, Stellenbosch University, Cape Town, South Africa; Division of Medical Microbiology, Faculty of Health Sciences, University of Cape Town, Cape Town, South Africa; Division of Medical Microbiology, Pathcare, Cape Town, South Africa; Division of Medical Microbiology, Ampath, Cape Town, South Africa; Division of Medical Microbiology, National Health Laboratory Service, Tygerberg Hospital, Cape Town, South Africa; Division of Medical Microbiology, Faculty of Medicine and Health Sciences, Stellenbosch University, Cape Town, South Africa; Division of Medical Microbiology, National Health Laboratory Service, Groote Schuur Hospital, Cape Town, South Africa; Division of Medical Microbiology, Faculty of Health Sciences, University of Cape Town, Cape Town, South Africa; Faculty of Health Sciences, Institute of Infectious Disease and Molecular Medicine, University of Cape Town, Cape Town, South Africa

## Abstract

**Background:**

Carbapenem-resistant Enterobacterales (CRE) are a substantial problem in Cape Town. CRE epidemiology is largely unknown and mortality remains high.

**Objectives:**

To describe and characterize the clinical and microbiological epidemiology of CRE within Cape Town hospitals to better inform therapy with regard to current and novel antibiotics, as well as improve antimicrobial stewardship (AMS), and infection prevention and control (IPC).

**Methods:**

This prospective, multicentre study performed between 1 November 2020 and 30 November 2022, across three public and three private hospitals included hospitalized participants with CRE from clinical cultures. Participant demographics, clinical information and microbiology results were collected and analysed.

**Results:**

Ninety percent of participants were from public hospitals. The age distribution ranged from 7 days to 88 years. Notable risk factors for CRE infection included recent exposure to antibiotics, medical devices and surgery. The most prevalent species was *Klebsiella pneumoniae.* However, a higher proportion of *Serratia marcescens* compared with previous reports was identified. The detected carbapenemases were *bla*_OXA-48-like_ (80%) and *bla*_NDM_ (11%). With the exception of amikacin (63%), tigecycline (65%), colistin (95%) and ceftazidime/avibactam (87%), susceptibility to antibiotics was low.

**Conclusions:**

This study identified common risk factors for CRE infection and generated a description of carbapenemase enzymes, species distribution and antibiograms, enabling a better understanding of CRE epidemiology. This provides insights into transmission patterns and resistance determinants of CREs, beneficial to informing data-driven regional patient management, AMS and IPC strategies.

## Introduction

In 2017, the WHO published a list of priority pathogens for which the development of effective antibiotics is urgently needed.^1^ Carbapenem-resistant Enterobacterales (CRE) are listed as critical pathogens and until the new β-lactam/β-lactamase inhibitor combinations (BLICs) became available, colistin was the last line of defence against these pathogens.^1,2^

In South Africa, carbapenemase-producing Enterobacterales emerged in 2011 with the detection of NDM and *Klebsiella pneumoniae* carbapenemase (KPC). The detection of oxacillinase-48 (OXA-48) and one of its variants, OXA-181, followed in 2012.^3,4^ National surveillance initially described a predominance of NDM (59%) followed by OXA-48 and its variants (29%), from 1503 CRE isolates between 2012 and 2015, mainly cultured from blood (25%) and urine (22%).^5^ Outbreaks of both large across-province and smaller institutional clonal outbreaks of OXA-181-producing *K. pneumoniae*, respectively, were also described between 2012 and 2016.^6,7^ Subsequent national surveillance of CRE bacteraemia between 2015 and 2018, from 895 isolates, demonstrated a shift in the predominant carbapenemase to OXA-48 and variants (52%), followed by NDM (34%).^8^ Recent national surveillance of CRE bacteraemia between 2019 and 2020 confirmed these findings in 1082 isolates, where the most common carbapenemase detected was still OXA-48 and variants (76.8%), although increasing in proportion. This was followed by NDM (21.1%).^9^ Whilst KPC^3^ was one of the first carbapenemases to be described in South Africa, similar to IMI-2,^10^ VIM,^6,11,12^ GES^6,12,13^ and IMP,^11,12^ they have infrequently been identified locally.

*K. pneumoniae* has consistently been the most frequent species isolated from national surveillance. However, the proportion of *K. pneumoniae* isolated has increased from 60% to 80% between 2012 and 2020. *Enterobacter cloacae* complex (6%–14%) and *Serratia marcescens* (5%–6%) have also commonly been isolated.^5,8,9^

The number of CRE causing infection is rapidly increasing, the epidemiology continues to evolve, and the high mortality associated with these pathogens has escalated the situation to not only a public health priority, but also a clinical emergency.^2,8,9^ Apart from a few institutional reports,^7,14^ the clinical, microbiological and molecular epidemiology of CRE in Cape Town is mostly unknown. Recently, marked increases in CRE have been noted at both public and private hospitals in the city, in line with the rest of South Africa. However, these increases in CRE have not been fully analysed, either with regard to transmission or genetic repertoire.

An in-depth understanding of the CRE epidemiology will allow for better-informed therapeutic interventions with regard to old and new antibiotics, as well as improved antimicrobial stewardship (AMS), by facilitating evidence-based empirical and directed therapy in our setting. Furthermore, effective infection prevention and control (IPC) interventions necessitate data-driven strategies.

Therefore, this study aims to address some of this need, by describing and characterizing the clinical, microbiological and molecular epidemiology of CRE within the Cape Town Metropole, across public and private hospitals.

## Methods

### Study design, setting and population

This was a prospective, multicentre study. Participants were recruited via convenience sampling between 1 November 2020 and 30 November 2022, from three participating public hospitals (Groote Schuur Hospital, Red Cross War Memorial Children’s Hospital and Tygerberg Hospital) and three participating private hospitals (Mediclinic Panorama, Netcare Christiaan Barnard Memorial Hospital, Netcare Blaauwberg Hospital) in Cape Town, Western Cape, South Africa. Further information about each hospital is available in Table S1 (available as Supplementary data at *JAC-AMR* Online).

#### Inclusion criteria

**Figure 1.**
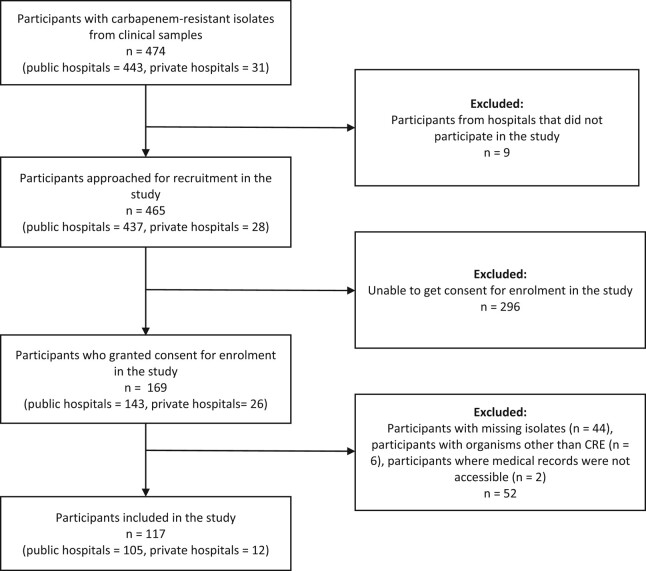
Flow diagram representation of participant inclusion in the study.

Hospitalized participants from whom phenotypic CRE (resistant to ertapenem, imipenem or meropenem) was isolated from clinical cultures obtained from any anatomical site during the study period (Figure 1).

#### Exclusion criteria

Duplicate or new CREs isolated from an already recruited participant (irrespective of whether the duplicate or new CRE was isolated from the participant during a subsequent admission to a different participating hospital), and participants with CRE isolated from surveillance cultures (rectal swabs, stool) were excluded. Enterobacterales with intrinsic reduced susceptibility to the carbapenems, such as *Proteus* spp., *Providencia* spp. and *Morganella* spp. with isolated imipenem resistance were not considered as CRE organisms, and participants from whom these organisms were cultured were also excluded.

### Bacterial identification and susceptibility testing methods

Bacterial isolates were cultured, identified and had routine susceptibility testing performed at microbiology laboratories serving the respective hospitals. Carbapenemase detection was also routinely performed at these laboratories with the RESIST-4 O.K.N.V. (Coris BioConcept, Belgium) lateral flow assay or the Xpert Carba-R (Cepheid, USA) PCR. These laboratories are accredited by the South African National Accreditation System and include two public laboratories [National Health Laboratory Service (NHLS) laboratories at Groote Schuur Hospital and Tygerberg Hospital] and two private laboratories (Pathcare and Ampath). The study group did not perform confirmatory testing of isolate identification, routine susceptibility results or carbapenemase results.

Identification of bacterial isolates was performed using the VITEK 2 automated system (bioMérieux, France) or the MALDI-TOF VITEK MS (bioMérieux) platform. Susceptibility testing was performed using the VITEK 2 automated system (bioMérieux), Kirby–Bauer disc diffusion, ETEST (bioMérieux) gradient diffusion, or broth microdilution methods where relevant. Results of antibiotic susceptibility tests were interpreted using the CLSI (NHLS and Pathcare laboratories) or the EUCAST (Ampath laboratories) guidelines for the relevant year. Ceftazidime/avibactam ETEST (bioMérieux) MIC and colistin broth microdilution MIC were performed on selected isolates by participating laboratories. When these specific MIC results were not available from participating laboratories, the study group referred the isolates to Ampath laboratories for testing.

### Informed consent

In-person or telephonic informed consent from participants or legal guardians, in the case of minors, was obtained. Similarly, informed consent was obtained from relatives when participants were too unwell to give consent, or had died.

### Data collection and statistical analysis

Participant demographics, clinical information (including outcome status within 90 days after discharge) and microbiology results of bacterial isolates were prospectively collected from both the participant and review of paper-based and electronic medical and laboratory reports. Data were collected on a participant questionnaire and folder review document and entered into an electronic database (REDCap) for analysis. Data were analysed using STATA version 14 (College Station, TX, USA).

A descriptive analysis was performed on the demographics and clinical epidemiology of participants and the microbiological characteristics of bacterial isolates. Categorical variables were summarized using proportions and percentages, and continuous variables were summarized as medians with IQRs. Univariable and multivariable logistic regression analysis was performed to investigate factors that may be associated with in-hospital mortality. A sample size estimate for the logistic regression analysis was not performed. Variables were selected *a priori* based on the likelihood to contribute to death. Variables were included in the multivariable analysis if the *P* value was ≤0.2 in the univariable analysis. A *P* value of 0.05 was regarded as statistically significant.

### Study definitions

The definition of MDR organisms (MDROs) included CRE, carbapenem-resistant *Acinetobacter baumannii*, carbapenem-resistant *Pseudomonas aeruginosa*, VRE and MRSA.

### Ethics

This study adhered to the principles outlined in the Declaration of Helsinki. Ethical approval for the study (HREC 096/2020), as well as for an isolate and clinical data biorepository (HREC R009/2020), was obtained from the University of Cape Town Human Research Ethics Committee. Ethical approval was also obtained from Stellenbosch University Health Research Ethics Committee as part of a reciprocal review (N21/01/002_RECIP_UCT_096/2020).

## Results

Clinical, epidemiological and microbiological data were collected from 117 patients during the study period (Table 1). Ninety percent (*n* = 105/117) of study participants were from three public hospitals, with the remainder from three private hospitals (10%, *n* = 12/117).

**Table 1.** Clinical and epidemiological characteristics of study participants

Variable	Participants	
	*n*	%
Gender (*N* = 117)		
Male	60	51
Female	57	49
Age (*N* = 117)		
<28 days (neonates)	6	5
<10 years	23	20
10–20 years	2	2
20–40 years	27	23
40–60 years	39	33
60–80 years	18	15
>80 years	2	2
Presence of comorbidities (*N* = 108)		
Yes^a^	55	51
No	53	49
Hospitalization in the year preceding positive CRE sample collection (*N* = 103)		
Yes^b^	45	44
No	58	56
Surgical history (*N* = 104)		
Yes^c^	88	85
No	16	15
Antibiotic exposure in the 30 days prior to positive CRE sample collection or current hospital admission (*N* = 109)		
Yes	108	99
No	1	1
Presence of medical device at any time from admission to positive CRE sample collection (*N* = 107)		
Yes^d^	95	89
No	12	11
Area of admission at the time of positive CRE sample collection (*N* = 109)		
ICU and high care unit	52	48
Wards^e^	57	53
Previous colonization or isolation of an MDR organism (*N* = 117)		
Yes^f^	39	33
No	78	67
Housing status (*N* = 114)		
Housing with indoor toilet and indoor water supply	98	86
Housing/informal shelter without indoor toilet and indoor water supply	16	14
Interprovince (interstate) or international travel in the 12 months prior to positive CRE sample collection (*N* = 110)		
Yes	4	4
No	106	96
In-hospital outcome (*N* = 115)		
Discharged	82	71
Died	33	29
Outcome of participant after discharge (*N* = 82)		
Readmission to hospital within 90 days after discharge	35	43
Died within 90 days after discharge	4	5

^a^HIV (*n* = 17), TB (*n* = 16), chronic kidney disease (*n* = 14), diabetes mellitus (*n* = 10), haematological malignancy (*n* = 10), chronic gastrointestinal (including liver and pancreas) disease (*n* = 8), cardiovascular disease (*n* = 7), chronic pulmonary disease (*n* = 7), hypertension (*n* = 3), cerebrovascular disease (*n* = 2), organ malignancy (*n* = 1), cryptococcosis (*n* = 1), recurrent brain abscess (*n* = 1), hypercholesterolaemia (*n* = 1).

^b^One previous admission to hospital (*n* = 19),  ≥ 2 previous admissions to hospital (*n* = 20), number of previous hospital admissions unknown (*n* = 6).

^c^Gastrointestinal surgery (*n* = 45), skin and soft-tissue debridement or drainage (*n* = 10), cardiothoracic surgery (*n* = 9), obstetric and gynaecological surgery (*n* = 2), neurosurgery (*n* = 1), other surgery (*n* = 21).

^d^Urine catheter (*n* = 76), central venous catheter (*n* = 65), nasogastric tube (*n* = 45), endotracheal tube (*n* = 36).

^e^Medical or surgical ward (*n* = 45), burns ward (*n* = 5), non-haematological oncology and transplant ward (*n* = 5), haematological oncology ward (*n* = 2).

^f^CRE (*n* = 28), carbapenem-resistant *Acinetobacter* species (*n* = 5), carbapenem-resistant *P. aeruginosa* (*n* = 4), MRSA (*n* = 2).

### Demographics and clinical characteristics

A description of the study population is presented in Table 1. Fifty-one percent (*n* = 60/117) were male participants. The age distribution ranged from 7 days to 88 years. The overall median age was 41 years (IQR 10–53). Children younger than 10 years accounted for 25% of the study population and the median age of this population was 122 days (IQR 43–676). The median time from admission to positive CRE sample collection was 15 days (IQR 8–35). In participants where data were available, 48% (*n* = 52/109) of participants were in the ICU at the time of positive CRE sample collection. Fifty-one percent (*n* = 55/108) had at least one underlying medical condition, of which the most common were HIV infection (31%, *n* = 17/55), TB (29%, *n* = 16/55) and chronic kidney disease (25%, *n* = 14/55). Forty-four percent of participants (*n* = 45/103) had previously been admitted to hospital, with 45% (*n* = 20/45) having at least two previous hospital admissions. Eighty-five percent (*n* = 88/105) of participants had at least one surgical procedure within 1 year prior to the positive CRE sample collection, with gastrointestinal tract surgery (51%, *n* = 45/88) being the most common. Almost all participants had received antibiotics within 30 days prior to their current admission or positive CRE sample collection (99%, *n* = 108/109). Most participants (89%, *n* = 95/107) had at least one medical device *in situ* at any time from admission to the time of positive CRE sample collection. Urine catheters (80%, *n* = 76/95) and central venous lines (68%, *n* = 65/95) were the most common. An MDRO had been previously isolated from thirty-three percent (*n* = 39/117) of participants, either from a clinical sample or from a surveillance sample, within the year prior to the current positive CRE sample. Gram-negative MDROs were the most common previously isolated (95%, *n* = 37/39) organisms, of which the most frequent was CRE (78%, *n* = 28/39). No VRE were previously isolated, but MRSA had been isolated from two patients (5%, *n* = 2/39) within the year prior to the current positive CRE sample. Most participants lived in formal housing with a plumbed water supply and indoor toilet (86%, *n* = 98/114) and had no interprovince (interstate) or international travel in the year prior to the positive CRE sample collection (96%, *n* = 106/110).

### Characteristics of CRE isolates

*Klebsiella* species were the predominant CRE isolates (74%, *n* = 87/117), with *K. pneumoniae* (*n* = 85) the most common, accompanied by one *Klebsiella oxytoca* isolate and one *Klebsiella variicola* isolate *(*identification confirmed by WGS), as outlined in Table 2. The other CRE species isolated included *S. marcescens* (11%, *n* = 13/117), *E. cloacae* (7%, *n* = 8/117), *Citrobacter freundii* (4%, *n* = 5/117), *Escherichia coli* (2%, *n* = 2/117) and *Providencia rettgeri* (2%, *n* = 2/117).

**Table 2.** Microbiology and antibiotic susceptibility of CRE isolates

Variable	*n*	%
CRE species isolated (*N* = 117)		
*Klebsiella* species^a^	87	74
*S. marcescens*	13	11
*E. cloacae*	8	7
*C. freundii*	5	4
*E. coli*	2	2
*P. rettgeri*	2	2
Carbapenemase (*N* = 79)		
*bla*_OXA-48-like_	63	80
*bla*_NDM_	9	11
Not detected	10	13
Sample type from which CRE was isolated (*N* = 117)		
Sterile	75	64
Blood culture	43	57
Tissue	12	16
Fluid	20	27
Non-sterile	42	36
Urine	21	50
Respiratory sample	9	21
Intraoperative pus/pus swab	10	24
Central venous catheter tip	2	5
Carbapenem MIC (mg/L)		
Ertapenem (*N* = 114)		
≤0.5	17	15
>0.5 and ≤8	32	28
>8	65	57
Imipenem (*N* = 117)		
≤1	42	36
>1 and ≤8	40	34
>8	35	30
Meropenem (*N* = 117)		
≤1	37	32
>1 and ≤8	31	26
>8	49	42
Colistin (*N* = 95)^b^		
≤2	90	95
≥4	5	5
Ceftazidime/avibactam (*N* = 99)^c^		
≤8/4	86	87
≥16/4	13	13

^a^ *K. pneumoniae* (*n* = 85), *K. oxytoca* (*n* = 1), *K. variicola* (*n* = 1).

^b^Excludes CRE with intrinsic resistance to colistin, determined by broth microdilution.

^c^Excludes NDM-producing CRE.

Carbapenemase testing was performed on 79 isolates (68%), and only *bla*_OXA-48-like_ (80%, *n* = 63/79) and *bla*_NDM_ (11%, *n* = 9/79) were detected. Three (4%, *n* = 3/79), of these isolates had both *bla*_OXA-48-like_ and *bla*_NDM_ detected. In 10 isolates (13%), no carbapenemase was detected.

**Figure 2.**
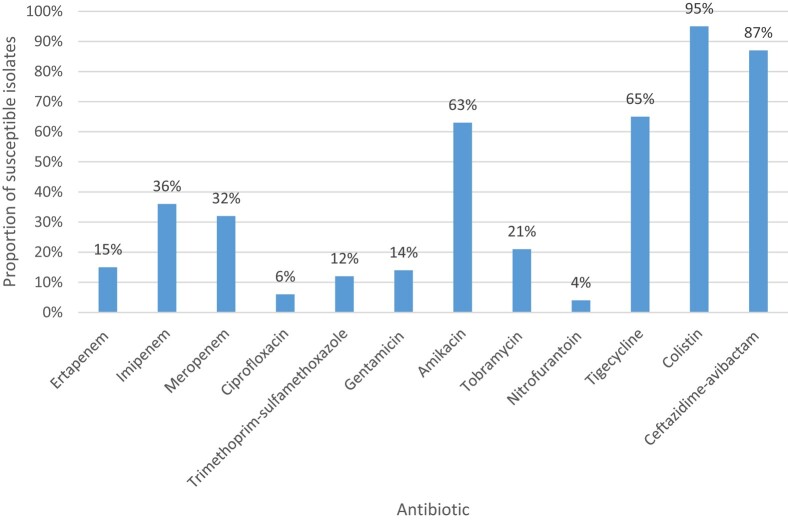
Antibiotic susceptibility of CRE expressed as proportion (%) susceptible.

Overall susceptibility to the non-β-lactam antibiotics was: tigecycline (excluding isolates with intrinsic resistance) 65% (*n* = 53/81); amikacin 63% (*n* = 71/113); gentamicin 14% (*n* = 16/115); tobramycin 21% (*n* = 6/29); ciprofloxacin 6% (*n* = 7/114); trimethoprim/sulfamethoxazole 12% (*n* = 14/115); nitrofurantoin 4% (*n* = 1/28) and colistin (excluding isolates with intrinsic resistance) 95% (*n* = 90/95). The median colistin MIC (*n* = 95) (excluding isolates with intrinsic resistance) was 1 mg/L (IQR 0.5–1). Susceptibility to ertapenem, imipenem and meropenem was 15% (*n* = 17/114), 36% (*n* = 42/117) and 32% (*n* = 37/117), respectively (Figure 2). The median ertapenem, imipenem and meropenem MIC was 32 mg/L (IQR 1–32), 2 mg/L (IQR 1–32) and 4 mg/L (IQR 0.5–32), respectively. Susceptibility to ceftazidime/avibactam (excluding NDM-producing isolates) was 87% (*n* = 86/99) and the median ceftazidime/avibactam MIC (*n* = 99) (excluding isolates with NDM carbapenemase production) was 1 mg/L (IQR 0.5–1). Of the non-NDM-producing ceftazidime/avibactam-resistant isolates (13%, *n* = 13/99), no carbapenemases were detected (*n* = 4), or the isolates were not tested for the presence of a carbapenemase (*n* = 9).

### Mortality and outcome

The all-cause in-hospital mortality rate was 29% (*n* = 33/115). The median time from positive CRE sample collection to death was 27 days (IQR 14–38).

For patients who were discharged (71%, *n* = 82/115), the median time from positive CRE sample collection to discharge was 19 days (IQR 11–39). Of the participants who were discharged, 43% (*n* = 35/82) were readmitted to hospital (all-cause) within 90 days from discharge, and 5% (*n* = 4/82) died (all-cause) within 90 days after discharge.

In the univariable logistic regression analysis, being in the ICU at the time of CRE sample collection, previous isolation of an MDRO and isolation of CRE from a sterile sample significantly increased the odds of in-hospital mortality. However, this significance was not confirmed by multivariable logistic regression analysis (Table 3).

**Table 3.** Univariable and multivariable analysis of variables associated with in-hospital mortality in hospitalized patients with CRE

Variable	Alive (*n*)	Died (*n*)	Unadjusted OR (95% CI)	*P* value	Adjusted OR (95% CI)	*P* value
Gender						
Male	42	16	Ref	—	NA	NA
Female	40	17	1.12 (0.50–2.5)	0.79		
Age (years)						
<20	24	7	Ref	—	NA	NA
20–40	18	9	1.71 (0.54–5.48)	0.36		
40–60	28	11	1.35 (0.45–4.02)	0.59		
60–80	11	5	1.56 (0.40–6.02)	0.52		
>80	1	1	3.43 (0.19–62.10)	0.40		
Presence of comorbidities						
No	38	15	Ref	—	NA	NA
Yes	38	16	1.07 (0.46–2.46)	0.88		
Hospitalization in the year preceding positive CRE sample collection						
No	41	16	Ref	—	NA	NA
Yes	35	9	0.66 (0.26–1.67)	0.38		
Surgical history						
No	12	3	Ref	—	NA	NA
Yes—gastrointestinal surgery	33	11	1.33 (0.32–5.61)	0.70		
Yes—non gastrointestinal	29	14	1.93 (0.47–7.96)	0.36		
Presence of medical device at any time from admission to positive CRE sample collection						
No	10	2	Ref	—	NA	NA
Yes	63	30	2.38 (0.49–11.55)	0.28		
Area of admission at the time of positive CRE sample collection (*N* = 109)						
Wards	45	12	Ref	—		
ICU and high care unit	32	18	2.11 (0.89–4.98)	0.09	0.88 (0.79–4.72)	0.15
Previous colonization or isolation of MDR organism						
No	60	16	Ref	—		
Yes	22	17	2.90 (1.25–6.71)	0.01	2.08 (0.84–5.15)	0.11
Sample type						
Non-sterile^a^	35	7	Ref	—		
Sterile^b^	47	26	2.77 (1.08–7.10)	0.03	2.40 (0.90–6.41)	0.08
Organism						
Non-*Klebsiella* species	22	7	Ref	—	NA	NA
*Klebsiella* species	60	26	1.36 (0.52–3.58)	0.53		

NA, not applicable.

^a^Urine, tracheal aspirate, sputum, pus/pus swab, central venous catheter tip.

^b^Blood culture, tissue, fluid.

## Discussion

Recent publications on the epidemiology of CRE bacteraemia from three public teaching hospitals in the Western Cape demonstrated an increase in the number and proportion of cases between 2015 and 2018 (7% *n* = 112/1601) and between 2019 and 2020 (14% *n* = 298/2144).^8,9^ This alarming situation in the Western Cape follows trends of rising global CRE rates.^15–17^

Similar to previous studies in South Africa,^5,8,9^ and globally,^15–17^ the most prevalent CRE organism in this study was *K. pneumoniae.* However, of concern is the higher proportion of *S. marcescens* identified in our study (11%), compared with what has previously been described in South Africa (5%–6%),^4,16^ as this organism is intrinsically resistant to colistin, further limiting therapeutic options. The only detected carbapenemases were *bla*_OXA-48-like_ and *bla*_NDM_, of which *bla*_OXA-48-like_ was the most prevalent. This is consistent with previous epidemiology suggesting that detection of *bla*_VIM_, *bla*_IMP_ and *bla*_KPC_ is uncommon in our setting.^5,8,9^ Differentiation of these enzymes is important to guide the use of ceftazidime/avibactam, which has recently been registered in South Africa. To preserve its longevity, updated country-specific recommendations are to steward its use within a framework that recognizes the current predominant resistance mechanisms in carbapenem-resistant Gram-negative bacteria.^2^

With the exception of amikacin, tigecycline and colistin, overall susceptibility to all other non-β-lactam antibiotics that were tested was low, in keeping with what has recently been described.^9^ In contrast, the proportion of isolates in this study with imipenem and meropenem MICs of >8 mg/L was higher than recently described (30% and 42% of isolates, respectively, compared with 23% and 36% of isolates, respectively) in national surveillance of CRE isolates causing bacteraemia from 2019 to 2020.^9^ This increase in the proportion of isolates with MICs of >8 mg/L is of serious clinical concern as this excludes imipenem and meropenem as potential treatment for CRE infections, where country-specific stewardship recommendations include them in select patients when the MICs are  ≤ 8 mg/L.^2^

Although colistin MICs were ≤2 mg/L in almost all isolates, similar to amikacin and tigecycline, colistin usage for CRE infection is limited due its side-effect profile, dosing and administration challenges, and questionable therapeutic efficacy.^2,18^ Notably, 13% of isolates (excluding NDM-producing isolates, *n* = 13/99) were resistant to ceftazidime/avibactam. In these isolates no carbapenemases were detected (*n* = 4) or tested for (*n* = 9). All tested isolates with only OXA-48-like carbapenemase detected (*n* = 55) were susceptible to ceftazidime/avibactam. Given that ceftazidime/avibactam has recently been registered in South Africa, and is only effective against specific resistance mechanisms, it is crucial that any gaps in knowledge around resistance-conferring mechanisms to novel antibiotics are identified with ongoing surveillance of susceptibility. Similarly, once the new siderophore cephalosporin cefiderocol becomes registered and available for use in South Africa, resistance and resistance-conferring mechanisms to this antibiotic will have to be monitored.

The most notable risk factors for CRE infection in this study included recent exposure to antibiotics, medical devices or surgery. Although AMS programmes have proved valuable in decreasing antibiotic consumption, antibiotic use in low- and middle-income countries is still comparatively higher than in high-income countries and improved context-based strategies to implement and optimize AMS are needed.^19^ Additionally, optimal management of medical devices (including reducing inappropriate use) should be effectively incorporated into AMS programmes.

Notably, 25% of the participants with CRE infections were children less than 10 years old. Whilst CRE infections are difficult to treat in adults, this is further exacerbated by limited safety, efficacy and dosing data in children for the ‘last-line antibiotics’ such as tigecycline and colistin and newer BLICs such as ceftazidime/avibactam.^18^ Additionally, the use of IPC measures such as contact precautions and isolation limits physical movement and social interactions with staff, carers, siblings and other children, and adversely impacts children.^20^ This highlights that current ‘adult-centric’ treatment options and IPC strategies are even more challenging with paediatric populations, especially in resource-constrained environments such as ours.

## Limitations

Whilst this study provided valuable insights into the epidemiology of CRE in Cape Town, there were several limitations and challenges. The impact of the SARS-CoV-2 pandemic on voluntary hospital participation and required approval processes, as well as participant recruitment, was substantial. This resulted in very few private hospitals volunteering to participate in the study. Additionally, participant recruitment was severely impacted due to additional IPC measures implemented in response to the pandemic, which included restriction of study-related research and movement of study-related-staff within participating institutions. Hence, whilst recruitment of participants started in 2020, only a limited number of participants were recruited in 2020 (3%, *n* = 4/117), with more participants recruited in 2021 (22%, *n* = 26/117) and most participants recruited in 2022 (74%, *n* = 87/117). Most of the participants were recruited from the large academic teaching public hospitals. The public hospitals included in the study comprised all three main academic teaching hospitals in Cape Town and the data are most representative of this sector. Therefore, the true burden of CRE in the private sector and within smaller non-academic public hospitals is under-represented in this study. Similarly, the impact of the additional IPC measures may have also influenced CRE transmission dynamics within hospitals, as well as patient travel within and outside of South Africa.

A sample size estimate for variables associated with all-cause mortality in the logistic regression analysis was not performed and may have led to under- or over-representation of this data.

Laboratory-related limitations include not confirming all isolate identification, susceptibility and carbapenemase results with a standardized methodology. Likewise, the usage of both CLSI and EUCAST for susceptibility interpretation, depending on which microbiology laboratory was used, reflects real-life practice in Cape Town. However, it is unlikely that this would have affected the reported antibiogram as most isolates were from laboratories where CLSI criteria are used routinely. Furthermore, routine carbapenemase testing resulted in only 68% of isolates being tested, again reflecting the impact of non-standardized and variable real-life laboratory processing and practices. The CRE incidence or prevalence rate was not calculated, which might have served as a better indicator to evaluate the impact of AMS and IPC interventions. Lastly, despite being a prospective study, due to our medical records and prescription charts predominantly being paper-based, missing data were inevitable and could have affected the demographic and clinical epidemiology, highlighting the need for fully integrated electronic medical record keeping systems.

### Conclusions

Despite the limitations, the study generated a description of carbapenemase enzymes and species distribution, enabling a better understanding of the epidemiology and risk factors for CRE. The information should inform data-driven regional patient management, AMS and IPC strategies, which could be beneficial to both public and private healthcare systems. Curtailing the spread and acquisition of CRE necessitates a comprehensive, coordinated and integrated approach including further studies of the determinants and rates of gastrointestinal colonization with CRE.

## Supplementary Material

dlae051_Supplementary_Data

## Members (collaborators) of the Cape Town Antimicrobial Stewardship Study Alliance (CT-ASSA)

Mediclinic Hospital group: Claudine Page, Elizabeth Schoeman, Elizma de Klerk, Karin Lategan, Karlien Pienaar, Liezl Henning, Mandy Du Plessis, Nomfundo Maseko, Salome Nel, Melenie Narainsamy, Michelle Vermeulen, Narissa du Toit, Teresa van Heerden. Netcare Hospital Group: Liza Sitharam, Asa Barendse, Dane Nagel, Jacqueline Prince, Letitia Vass, Rileen Strauss, Rushana Fakier. Pathcare Laboratory group: Catherine Samuel, Marelieze van Zyl, Leigh-Ann Isaacs, Shareefa Hendricks, Amy Dodd, Reecka Daniels. University of Cape Town Medical Microbiology Group: Widaad Zemanay, Judi Van Heerden, Nchimunya Hapeela, Parveen Brown, Zubayr Daniels, Shantelle Claassen, Fadheela Patel. Life Healthcare Hospital Group: Sharon Vasuthevan, Enid Scott, Esmeralda Ricks, Patricia Curle. Lancet Laboratory Group: Justyna Wojno.

## Supplementary data

Table S1 is available as Supplementary data at *JAC-AMR* Online.
