# Supplementary material for: Carbapenem-resistant Enterobacterales among hospitalized patients in Cape Town, South Africa: clinical and microbiological epidemiology
Source: JAC Antimicrob Resist. 2024 Mar 22;6(2):dlae051. doi: 10.1093/jacamr/dlae051 (PMC10959510; doi:10.1093/jacamr/dlae051)
Supplement: dlae051_Supplementary_Data [file dlae051_supplementary_data.docx]

Supplementary material Table 1: Characteristics of hospitals included in the study

| Hospital | Population | Category | Bed number | Speciality services | Infection prevention and control / antimicrobial stewardship program^a^ |
| --- | --- | --- | --- | --- | --- |
| Groote Schuur Hospital | Adult  Paediatric | Public Predominantly referral | 950 | Medical (including HIV), Surgical (including transplants), Maternal, Paediatrics (including neonatology), Mental health, Oncology, Haematology | Yes |
| Tygerberg Hospital | Adult  Paediatric | Public Predominantly referral | 1384 | Medical (including HIV), Surgical (including transplants), Maternal, Paediatrics (including neonatology), Mental health, Oncology, Haematology | Yes |
| Red Cross War Memorial Children’s Hospital | Paediatric | Public Predominantly referral | 272 | Paediatric medical (including HIV), Paediatric surgical (including transplants and neonatal surgery), Paediatric oncology, Paediatric haematology | Yes |
| Mediclinic Panorama Hospital | Adult  Paediatric | Private Community and Referral | 400 | Medical (including HIV), Surgical, Maternal, Paediatrics (including neonatology), Mental health, Oncology, Haematology | Yes |
| Netcare Christiaan Barnard Hospital | Adult  Paediatric | Private Community and Referral | 248 | Medical (including HIV), Surgical (including transplants), Maternal, Paediatrics (including neonatology), Mental health, Oncology, Haematology | Yes |
| Netcare Blaauwberg Hospital | Adult  Paediatric | Private Community and Referral | 100 | Medical (including HIV), Surgical, Maternal, Paediatrics (including neonatology), Mental health, Oncology, Haematology | Yes |

^a^ Includes a range of antimicrobial stewardship ward rounds and/or prospective audit and feedback and/or antibiotic utilization surveys and/or peri-operative antibiotic prophylaxis interventions and/or use of antibiotic guidelines
